# MRI evaluation of Pacinian corpuscle number and distribution in the forefoot in diabetic sensorimotor polyneuropathy

**DOI:** 10.1186/s13244-025-01932-8

**Published:** 2025-03-07

**Authors:** Sophia S. Goller, Georg C. Feuerriegel, Adrian A. Marth, Christoph Germann, Martin Schubert, Michèle Hubli, Felix W. A. Waibel, Reto Sutter

**Affiliations:** 1 Department of Radiology, Balgrist University Hospital, Faculty of Medicine, University of Zurich, Zurich, Switzerland; 2 Swiss Center for Musculoskeletal Imaging, Balgrist Campus AG, Zurich, Switzerland; 3 Spinal Cord Injury Center, Balgrist University Hospital, Faculty of Medicine, University of Zurich, Zurich, Switzerland; 4 Department of Orthopaedics, Balgrist University Hospital, Faculty of Medicine, University of Zurich, Zurich, Switzerland

**Keywords:** Pacinian corpuscle, MRI, Diabetes, Diabetic sensorimotor polyneuropathy

## Abstract

**Objectives:**

To evaluate Pacinian corpuscles (PC) in the forefoot of patients with type 2 diabetes-derived sensorimotor polyneuropathy (DSP) with MRI.

**Materials and methods:**

This single-center study compared 20 DSP patients who underwent clinical forefoot 3-T MRI to healthy volunteers matched for age and gender. Two radiologists independently assessed the number and distribution of PC. In addition, one radiologist determined PC size. Correlations between PC number, duration of diabetes, and Hemoglobin A1c (HbA1c) were assessed.

**Results:**

In DSP patients, the number of PC in the forefoot was significantly reduced compared to healthy volunteers (82.7 ± 46.1 vs. 265.3 ± 49.3, *p* < 0.001). In contrast to the typical “chain-like” pattern of PC in healthy volunteers, their arrangement was heterogeneous in DSP patients and showed a more isolated “spot-like” pattern. Volunteers exhibited the highest PC number along the proximal phalanges, followed by the metatarsophalangeal (MTP) joints, while in patients, no such predominance was found. In DSP patients, the maximum diameter of PC was 3 mm (range 1–3 mm) compared to 5 mm (1–5 mm) in healthy volunteers. In patients, the mean duration of diabetes was 234.8 ± 130.4 months, and the mean HbA1c was 7.6 ± 1.1%. There was no significant correlation between PC number, duration of diabetes, and HbA1c.

**Conclusion:**

DSP patients had threefold lower PC numbers in the forefoot and exhibited a “spot-like” PC distribution pattern rather than the typical “chain-like” pattern observed in healthy volunteers. The exact depiction of PC and their distribution in the forefoot opens up the possibility of using MRI as a noninvasive diagnostic tool to assess DSP.

**Critical relevance statement:**

MRI may serve as a noninvasive diagnostic tool for assessing patients with diabetic sensorimotor polyneuropathy as it allows for evaluating Pacinian corpuscle number and distribution in the forefoot.

**Key Points:**

- DSP patients showed three times lower forefoot PC numbers than healthy volunteers.
- PC distribution was altered in DSP patients and termed a “spot-like” pattern.
- Reduced PC (*n* < 9 along each MTP joint II-V) might be suspicious for DSP.

**Graphical Abstract:**

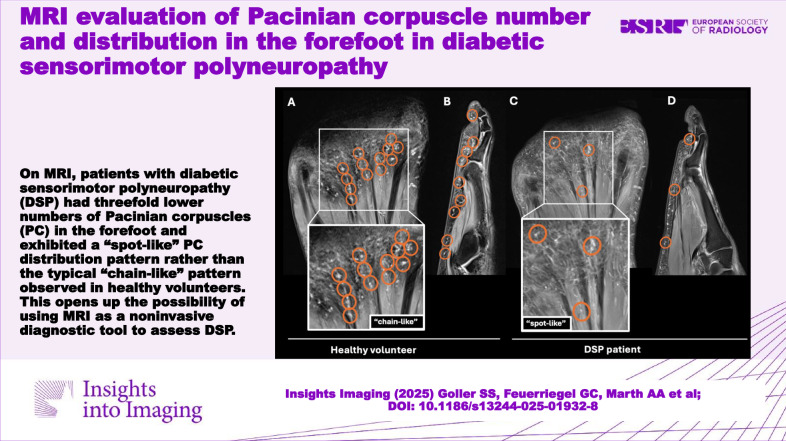

## Introduction

Pacinian corpuscles (PC), also known as Vater-Pacini or lamellar corpuscles, are located at the junction of the dermis and hypodermis, adjacent to and within capsuloligamentous structures, and are the primary sensory receptors for vibration and deep pressure besides Meissner corpuscles and Merkel cell-axonal complexes [1].

These sensory receptors are found all over the body but are particularly common on the hands and feet, occurring in groups of 3 to 5 per cm^2^ [2]. PC feature an oval shape and consist of single non-myelinated terminal nerve endings and their associated Schwann-derivate cells, surrounded on the outside by up to 60 layers of modified fibroblasts. Each stratum is separated from the next by a small fluid-filled space. In turn, the inner layers are surrounded by about 30 less dense modified fibroblastic layers surrounded by connective tissue, forming a capsule around a core. In the histological section, this lamellation gives PC the appearance of a sliced onion, and when pressure is put on the hands and feet, the deformation of these lamellae induces the action potential in the corresponding axon [3].

Diabetic sensorimotor polyneuropathy (DSP) is the most common complication of type 2 diabetes, affecting up to 50% of patients, and, in turn, 50% of those affected develop neuropathic pain [4–6]. Besides patient history and clinical examination, various tests based on a quantitative structural analysis of different nerve fibers, such as skin biopsy or confocal corneal microscopy, are available to diagnose neuropathies and determine their severity [7–11]. Besides deteriorated pain sensation, impaired touch and vibration perception are found in DSP, mainly mediated by Aβ-fibers and mechanoreceptors, including the PC [4–6, 12]. Further, in DSP, topographical and structural changes of different sensory receptors, including PC, have been proven [4, 13]. Although it has been shown that PC are visualizable with high-resolution ultrasound and MRI at various field strengths [14–17], no study has yet investigated macroscopic changes of PC in DSP patients using imaging.

Given this background, we used MRI to evaluate the number and distribution of PC in the forefoot of type 2 diabetes patients with neurologically proven DSP compared to healthy volunteers matched for age and gender.

## Materials and methods

### Study design and participants

This combined retrospective and prospective single-center study was approved by the local institutional review board (Cantonal Ethics Committee Zurich) and conducted according to national ethical standards and in adherence to the principles of the Declaration of Helsinki and its subsequent amendments.

### Patients

**Fig. 1:**
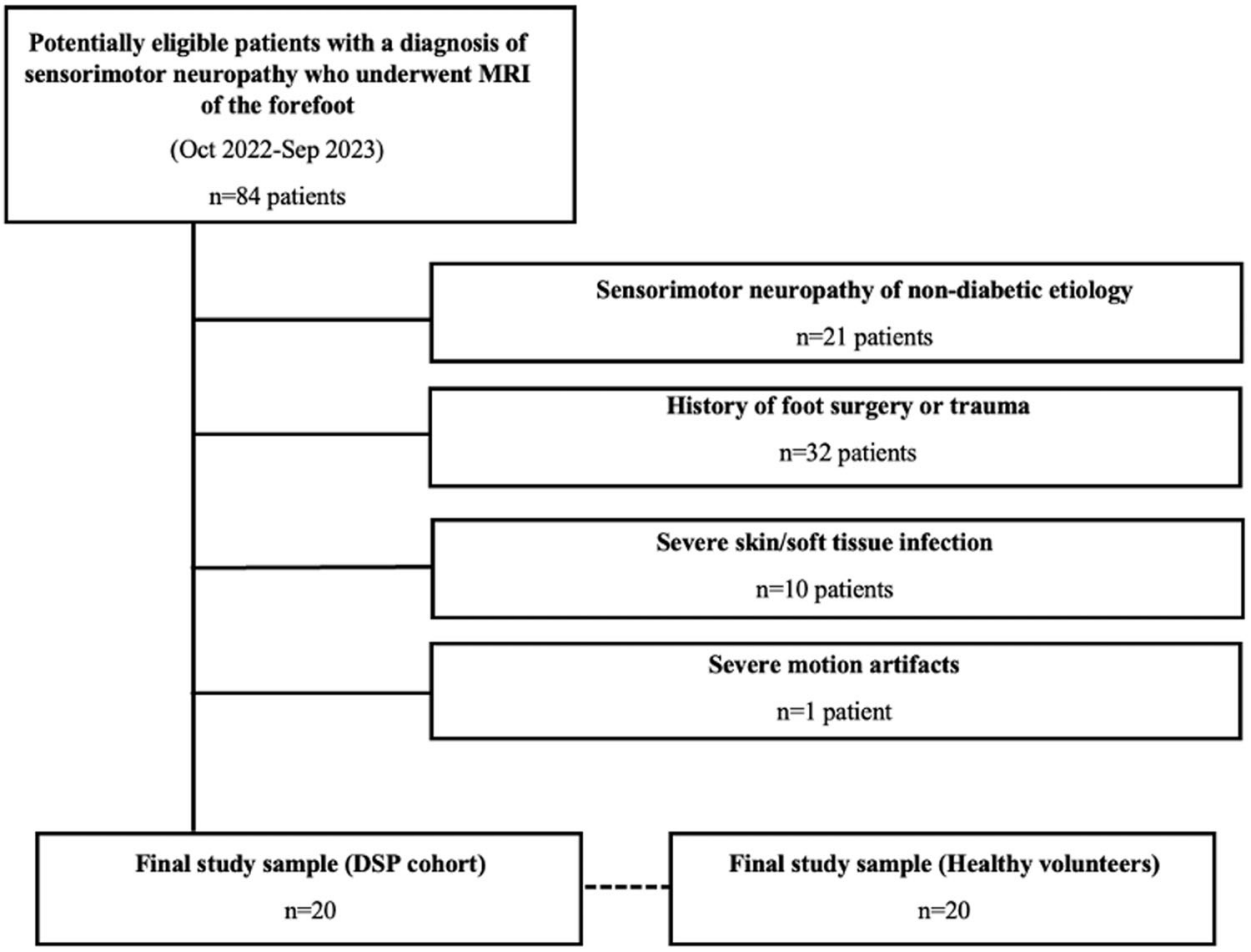
Flowchart illustrating the patient selection process and the study setup. Of 84 potentially eligible patients with a diagnosis of sensorimotor neuropathy who underwent clinical 3-T MRI of the forefoot, 64 were excluded during the selection process. This resulted in a study sample of 20 patients in the diabetic sensorimotor polyneuropathy (DSP) cohort and 20 healthy volunteers matched for age and gender who prospectively underwent 3-T MRI of the forefoot

All patients retrospectively included in the study had given written informed consent that allowed their health-related data to be used for scientific purposes. Patient charts were retrospectively reviewed for patients with type 2 diabetes and a neurologically confirmed diagnosis of DSP. According to current guidelines, DSP was diagnosed by neurological examination, including examinations on pain, temperature, touch and vibratory symptoms, and electroneurography [18]. Patients were eligible if they underwent clinical routine forefoot 3-T MRI between October 2022 and September 2023 (*n* = 20, Fig. 1). Patients with sensorimotor neuropathy of non-diabetic etiology, previous surgery or trauma, severe skin or soft tissue infection, or severe motion artifacts were excluded. The duration of type 2 diabetes and the last available Hemoglobin A1c (HbA1c) value were extracted from the patient charts.

### Healthy volunteers

Prospectively enrolled healthy volunteers provided written informed consent before the study. The cohort comprised healthy volunteers matched for age (± 10 years) and gender without evidence of neuropathy according to the Toronto Clinical Neuropathy Score, a clinical score incorporating sensory and motor symptoms and lower-limb sensory and reflex testing [19] (*n* = 20, Fig. 1). For healthy volunteers, further eligibility criteria included no history of diabetes (type 1 or 2) or other metabolic disease, previous surgery or trauma on the foot to be examined (same side according to the matched patient), and no contraindication to undergo MRI at 3-T field strength.

### Imaging protocol

The DSP cohort underwent clinically indicated MRI of the forefoot (the scan area comprised the Lisfranc joint line proximally and the toe tips distally) on a 3-T scanner (Magnetom Vida, Siemens Healthineers) with a dedicated 16-channel foot coil. Indications for imaging the patients comprised suspected infection (*n* = 17), suspected insufficiency fracture (*n* = 1), suspected Morton’s neuroma (*n* = 1), and suspected osteoarthrosis (*n* = 1). All patients were examined using a routine forefoot imaging protocol, including coronal and sagittal fat-saturated (fs) proton-density (PD)-weighted, sagittal and transversal T1-weighted, and transversal fs, as well as non-fs T2-weighted turbo spin-echo (TSE) sequences (detailed scan parameters are given in Table 1).

**Table 1:** MR imaging protocol of the forefoot

Sequence	Cor PD fs TSE	Sag PD fs TSE	Sag T1 TSE	Tra T1 TSE	Tra T2 TSE	Tra T2 fs TSE
Echo time (ms)	32	33	12	11	71	67
Repetition time (ms)	3500	4510	680	700	4500	5000
Bandwidth (Hz/px)	302	302	266	260	248	246
Acquisition matrix	448 × 347	448 × 246	448 × 246	400 × 288	448 × 325	384 × 278
Slice thickness (mm)	3	3	3	3	3	3
Slice number	20	34	34	24	24	24
FOV (mm)	158 × 163	160 × 110	159 × 110	117 × 130	117 × 130	117 × 130
Acquisition time (min:s)	1:20	1:34	0:53	0:36	0:49	2:05

*Cor* coronal, *FOV* field of view, *fs* fat-saturated, *Hz* Hertz, *PD* proton-density (-weighted), *px* pixel, *sag* sagittal, *tra* transversal, *TSE* turbo spin-echo

Healthy volunteers were scanned at another 3-T unit (Magnetom Prisma, Siemens Healthineers) using a dedicated 16-channel foot coil and the same protocol (Table 1).

### Image analysis

Image analysis was done on a Picture Archiving and Communication System (PACS) workstation (Merlin, Phoenix-PACS). All MRI studies were anonymized and independently reviewed by two musculoskeletal fellowship-trained radiologists (S.S.G. and G.C.F., with both 5 years of experience, respectively). Examinations were analyzed randomly, and both readers were blinded to clinical data and the results of the other reader. In uncertain cases, each reader had to determine independently and without external help from a third party whether the present image finding was a PC. To calculate intra-reader reliability, readings were repeated in the same fashion after 6 months.

**Fig. 2:**
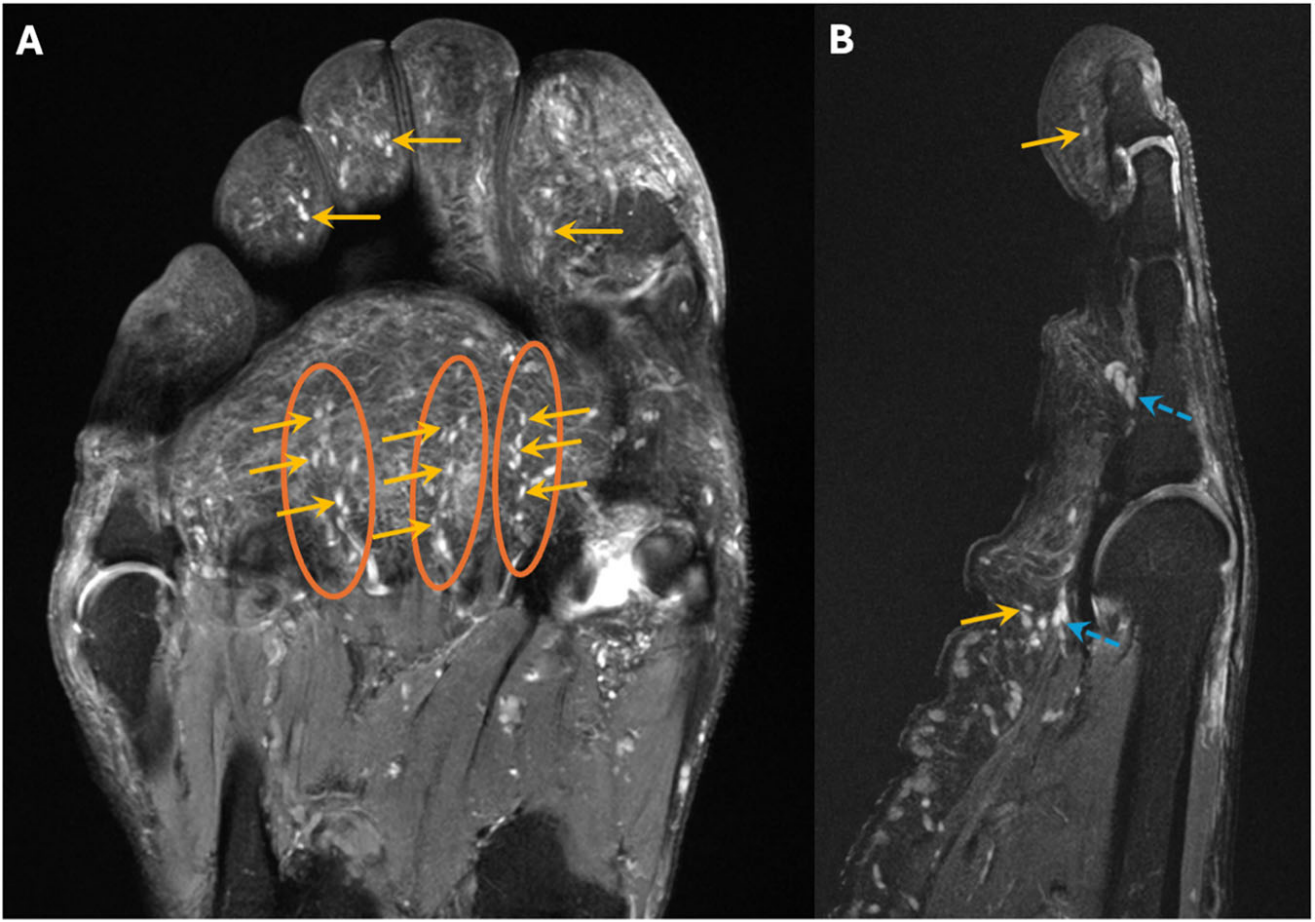
Normal MR-anatomy of Pacinian corpuscles (PC) of a healthy subject on forefoot MRI. Coronal (**A**) and sagittal (at the level of the fourth digit) (**B**) proton-density (PD)-weighted fat-saturated (fs) images of the right forefoot of a 60-year-old healthy male volunteer demonstrate numerous homogenously hyperintense ovoid nodules in the plantar subcutaneous fat (yellow arrows, **A**, **B**) and along the deep muscle fascia (dashed blue arrows, **B**), which are identified as PC. In the areas with the most PC, these are predominantly arranged in characteristic “chain-like” patterns, as best seen plantar to the metatarsophalangeal joints and proximal phalanges (circles, **A**)

**Fig. 3:**
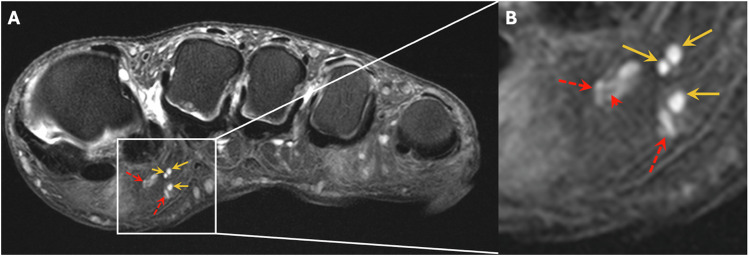
Differentiation of Pacinian corpuscles (PC) from blood vessels. Transversal proton-density (PD)-weighted fat-saturated (fs) image (**A**) of the foot of the same volunteer as in Fig. 2 at the level of the metatarsophalangeal joints. **B** illustrates a magnified area of **A**. Clustered PC (yellow arrows, **A**, **B**) are depicted in the plantar subcutaneous fat near a small vascular plexus with sections of tubular-shaped blood vessels (dashed red arrows, **A**, **B**), which are less hyperintense and have a more inhomogeneous signal than the homogenously hyperintense PC. In cross-section, some blood vessels appear as central hypointense and peripheral mildly hyperintense structures (red arrowhead, **B**). Additionally, blood vessels were confirmed by following their linear branching morphology over consecutive slices

The number of PC was separately assessed for two distinct localizations in relation to the skin and deep soft tissues within the forefoot. The first localization was designated as “(sub)cutaneous,” meaning PC located at the junction of the dermis and hypodermis, respectively, in the subcutaneous fat. The second localization was defined as “deep”, meaning PC located along the deep muscle fascia respectively adjacent to capsuloligamentous structures (Fig. 2). Readers assessed the number of PC at these two localizations for the following subregions for each digit: Distal phalanx (I-V), middle phalanx (II-V), proximal phalanx (I-V), metatarsophalangeal (MTP) joint (I-V), and metatarsal bone (I-V). According to Germann et al, two criteria were applied to differentiate PC from blood vessels, which were the linear and branching morphology of blood vessels ensured by following their course over consecutive slices and a central hypointensity with a peripheral hyperintense rim of blood vessels on cross-section as opposed to the homogenously hyperintense PC [14] (Fig. 3). Furthermore, one reader (S.S.G.) assessed the diameter of the PC in both groups. The PC were viewed in all three spatial planes and measured in the spatial plane where the maximum diameter was visible.

### Statistical analysis

Statistical analyses were performed in SPSS Statistics (v. 29, IBM Corporation). Quantile-Quantile plots and the Shapiro–Wilk test were used to test for normal distribution of continuous variables. Unless otherwise stated, nominal data is given as frequency with percentage in parentheses, while continuous data is presented as mean ± standard deviation. In addition to descriptive statistics, the Wilcoxon signed-rank test was used to evaluate differences in PC numbers between groups. Spearman’s rank correlation coefficients were used to measure the strength and direction of association between the variables “PC number”, “duration of diabetes”, and “HbA1c value”, respectively. The inter- and intra-reader agreement was analyzed using intraclass correlation coefficients (ICC). The level of agreement was reported as follows [20]: 0.0 = poor, 0.01–0.20 = slight, 0.21–0.40 = fair, 0.41–0.60 = moderate, 0.61–0.80 = substantial, > 0.80 = almost perfect. All statistical tests were performed two-sided, and a level of significance (α) below 0.05 was used. An a priori power analysis was conducted using G*Power (v. 3.1) [21, 22] to determine the minimum sample size required to test the study hypothesis. Results indicated the required sample size to be *n* = 13 to achieve a power of 0.80 for detecting a medium effect for the Wilcoxon signed-rank test.

## Results

### Demographics

The study included 20 DSP patients (mean age 67 ± 9.3 years, 15 males) and 20 healthy volunteers matched for age and gender (mean age 62 ± 9.9 years). Table 2 summarizes general demographic and clinical data of the DSP cohort and of healthy volunteers.

**Table 2:** Characteristics of patients with diabetic sensorimotor polyneuropathy (DSP) and healthy volunteers

	DSP patients	Healthy volunteers
Male sex	15 (75.0)	15 (75.0)
Age (years)	67 ± 9.3	62 ± 9.9
Height (cm)	177.1 ± 9.8	176.0 ± 8.8
Weight (kg)	95.9 ± 15.0	79.6 ± 14.8
Body mass index	30.6 ± 4.5	25.5 ± 3.6

Nominal data is given as frequency with percentage in parentheses. Continuous data are presented as mean ± standard deviation

*DSP* diabetic sensorimotor polyneuropathy

### Pacinian corpuscle number, distribution, and size

**Fig. 4:**
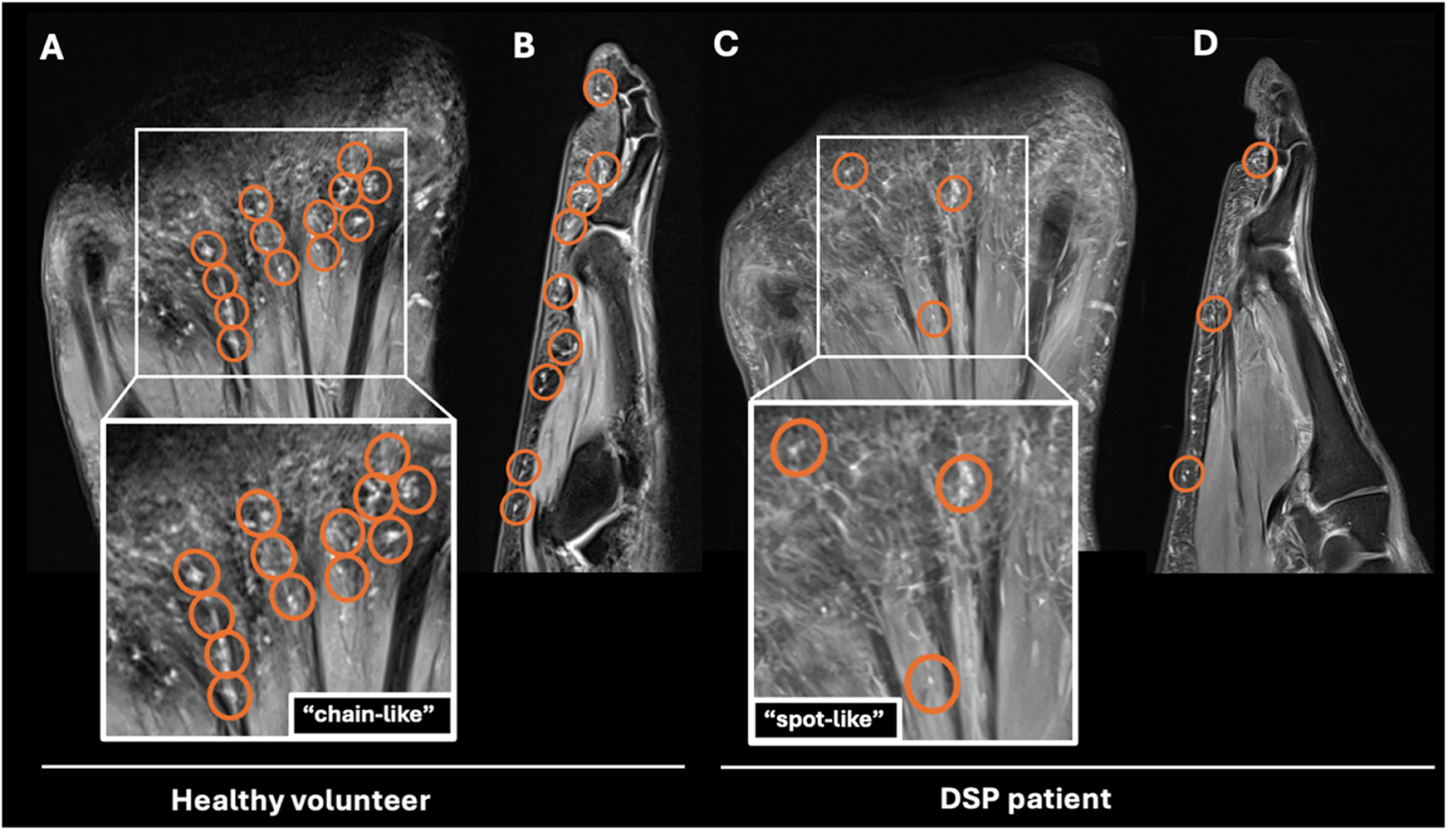
Rarefaction and disturbed arrangement of Pacinian corpuscles (PC) on forefoot MRI of a patient with diabetic sensorimotor polyneuropathy (DSP) compared to a healthy volunteer. Healthy volunteer: The coronal (**A**) and sagittal (at the level of the fourth digit) (**B**) proton-density (PD)-weighted fat-saturated (fs) images of the right forefoot of another 60-year-old healthy male volunteer depict numerous PC in the subcutaneous fat and along the deep muscle fascia (circles, **A**, **B**) showing a typical “chain-like” pattern. DSP patient: The coronal (**C**) and sagittal (at the level of the fourth digit) (**D**) PD-weighted fs images of the right forefoot of a 61-year-old male DSP patient who was diagnosed with type 2 diabetes 13 years ago demonstrate a significant rarefaction of PC numbers in the subcutaneous fat and along the deep muscle fascia and an altered arrangement with destruction of the typical “chain-like” pattern and clustering (circles, **C**, **D**). This was the predominant pattern seen in DSP patients, which we termed a “spot-like”-pattern

**Fig. 5:**
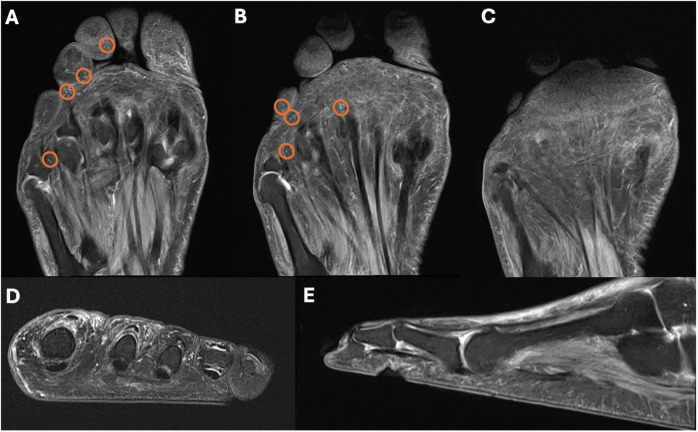
Severe rarefaction and disturbed arrangement of Pacinian corpuscles (PC) on forefoot MRI of a patient with diabetic sensorimotor polyneuropathy (DSP). Consecutive coronal proton-density (PD)-weighted fat-saturated (fs) images (**A**–**C**), axial T2-weighted fs image (at the level of the proximal phalanges) (**D**), and sagittal PD-weighted fs (at the level of the third digit) (**E**) image of the left foot of a 66-year-old male with DSP, who was diagnosed with type 2 diabetes 8 years ago. MRI demonstrates a severe reduction of PC numbers in the subcutaneous fat and along the deep muscle fascia, as well as an altered PC distribution (“spot-like” pattern): Only a few randomly distributed PC are visible in the plantar subcutaneous fat (circles **A**, **B**). As of note, these all had a small diameter (1–2 mm)

The overall number of PC in the forefoot of DSP patients was significantly reduced compared to healthy volunteers (mean ± standard deviation: 82.7 ± 46.1 vs. 265.3 ± 49.3, *p* < 0.001). Moreover, this was also evident for individual subregions for each digit (*p* ≥ 0.013) (Table 3, Figs. 4 and 5). For those PC located at the second to fifth MTP joint, the lowest overall number of PC in healthy volunteers was *n* = 9, whereas the highest number of PC in DSP patients was *n* = 6, i.e., the second to fifth MTP joints were an anatomical area with no overlap between the two groups. For the PC located at the first MTP joint, there was some overlap in the range of PC numbers, with a maximum PC number of *n* = 13 in DSP patients and a minimum PC number of *n* = 9 in healthy volunteers (Table 3), even though the mean number of PC was significantly different also for the first MTP joint (3.6 in DSP patients vs. 16.6 in healthy volunteers, *p* < 0.001).

**Table 3:** Number of Pacinian corpuscles for distinct localizations for each digit I-V

		DSP patients	Healthy volunteers	*p*-value^a^
**Digit I**	**Distal phalanx**			
	Overall	4.7 (1–11)	9.2 (6–13)	**< 0.001**
	(Sub)cutaneous	2.8 (0–5)	4.3 (3–6)	**< 0.001**
	Deep	1.9 (0–8)	4.9 (2–7)	**< 0.001**
	**Proximal phalanx**			
	Overall	5.8 (0–12)	17.4 (10–23)	**< 0.001**
	(Sub)cutaneous	3.4 (0–6)	8.3 (5–11)	**< 0.001**
	Deep	2.5 (0–7)	9.1 (5–12)	**< 0.001**
	**MTP joint**			
	Overall	3.6 (0–13)	16.6 (9–22)	**< 0.001**
	(Sub)cutaneous	1.9 (0–7)	8.4 (5–11)	**< 0.001**
	Deep	3.0 (0–7)	8.2 (4–11)	**< 0.001**
	**Metatarsal**			
	Overall	5.4 (0–12)	8.4 (5–13)	**0.001**
	(Sub)cutaneous	3.0 (0–8)	4.4 (3–8)	**0.002**
	Deep	2.4 (0–6)	4.0 (2–7)	**< 0.001**
**Digit II**	**Distal phalanx**			
	Overall	3.6 (0–7)	7.2 (5–10)	**< 0.001**
	(Sub)cutaneous	2.3 (0–5)	4.0 (3–6)	**< 0.001**
	Deep	1.3 (0–4)	3.3 (2–5)	**< 0.001**
	**Middle phalanx**			
	Overall	2.6 (0–7)	5.4 (3–8)	**< 0.001**
	(Sub)cutaneous	1.6 (0–4)	3.1 (1–5)	**< 0.001**
	Deep	1.0 (0–3)	2.3 (1–4)	**< 0.001**
	**Proximal phalanx**			
	Overall	3.8 (0–9)	17.9 (9–25)	**< 0.001**
	(Sub)cutaneous	2.5 (0–7)	13.0 (5–18)	**< 0.001**
	Deep	1.4 (0–4)	4.9 (3–7)	**< 0.001**
	**MTP joint**			
	Overall	2.0 (0–4)	15.9 (9–23)	**< 0.001**
	(Sub)cutaneous	1.3 (0–3)	9.7 (5–14)	**< 0.001**
	Deep	0.8 (0–2)	6.2 (4–9)	**< 0.001**
	**Metatarsal**			
	Overall	3.6 (0–11)	8.7 (5–13)	**< 0.001**
	(Sub)cutaneous	2.6 (0–8)	4.4 (2–9)	**< 0.001**
	Deep	1.0 (0–3)	4.3 (2–6)	**< 0.001**
**Digit III**	**Distal phalanx**			
	Overall	3.8 (0–11)	7.7 (4–13)	**< 0.001**
	(Sub)cutaneous	2.7 (0–7)	4.2 (2–8)	**< 0.001**
	Deep	1.1 (0–4)	3.5 (1–5)	**< 0.001**
	**Middle phalanx**			
	Overall	2.5 (0–7)	5.9 (3–9)	**< 0.001**
	(Sub)cutaneous	1.4 (0–4)	3.2 (1–5)	**< 0.001**
	Deep	1.1 (0–4)	2.7 (1–4)	**< 0.001**
	**Proximal phalanx**			
	Overall	3.7 (0–8)	17.3 (10–25)	**< 0.001**
	(Sub)cutaneous	2.4 (0–5)	8.3 (3–12)	**< 0.001**
	Deep	1.4 (0–3)	9.1 (6–13)	**< 0.001**
	**MTP joint**			
	Overall	1.9 (0–6)	16.3 (12–21)	**< 0.001**
	(Sub)cutaneous	1.2 (0–4)	7.8 (6–10)	**< 0.001**
	Deep	0.8 (0–2)	8.5 (6–11)	**< 0.001**
	**Metatarsal**			
	Overall	3.6 (0–11)	6.9 (4–11)	**< 0.001**
	(Sub)cutaneous	2.5 (0–7)	4.5 (3–7)	**< 0.001**
	Deep	1.2 (0–4)	2.4 (1–4)	**0.002**
**Digit IV**	**Distal phalanx**			
	Overall	4.3 (0–8)	7.6 (5–11)	**< 0.001**
	(Sub)cutaneous	3.2 (0–6)	4.8 (3–7)	**< 0.001**
	Deep	1.1 (0–3)	2.8 (2–4)	**< 0.001**
	**Middle phalanx**			
	Overall	1.7 (0–5)	5.4 (2–8)	**< 0.001**
	(Sub)cutaneous	1.1 (0–3)	3.0 (1–4)	**< 0.001**
	Deep	0.6 (0–3)	2.4 (1–4)	**< 0.001**
	**Proximal phalanx**			
	Overall	3.5 (0–8)	17.2 (11–24)	**< 0.001**
	(Sub)cutaneous	2.3 (0–6)	8.0 (4–11)	**< 0.001**
	Deep	1.3 (0–4)	9.2 (7–13)	**< 0.001**
	**MTP joint**			
	Overall	2.7 (0–6)	15.8 (11–20)	**< 0.001**
	(Sub)cutaneous	1.6 (0–4)	8.1 (6–10)	**< 0.001**
	Deep	1.1 (0–2)	7.7 (5–10)	**< 0.001**
	**Metatarsal**			
	Overall	4.5 (0–11)	7.5 (3–13)	**0.001**
	(Sub)cutaneous	2.9 (0–8)	4.7 (2–9)	**< 0.001**
	Deep	1.6 (0–5)	2.8 (0–5)	**0.004**
**Digit V**	**Distal phalanx**			
	Overall	4.2 (0–9)	8.0 (5–12)	**< 0.001**
	(Sub)cutaneous	3.1 (0–7)	4.8 (3–8)	**< 0.001**
	Deep	1.1 (0–3)	3.2 (2–5)	**< 0.001**
	**Middle phalanx**			
	Overall	1.7 (0–5)	4.8 (2–7)	**< 0.001**
	(Sub)cutaneous	1.0 (0–3)	2.7 (1–4)	**< 0.001**
	Deep	0.7 (0–2)	2.1 (1–3)	**< 0.001**
	**Proximal phalanx**			
	Overall	3.4 (0–8)	15.7 (8–22)	**< 0.001**
	(Sub)cutaneous	1.9 (0–4)	8.1 (2–12)	**< 0.001**
	Deep	1.6 (0–5)	7.7 (5–10)	**< 0.001**
	**MTP joint**			
	Overall	2.3 (0–6)	16.6 (11–22)	**< 0.001**
	(Sub)cutaneous	1.8 (0–5)	8.6 (6–12)	**< 0.001**
	Deep	1.0 (0–2)	8.0 (5–10)	**< 0.001**
	**Metatarsal**			
	Overall	4.3 (0–10)	6.6 (2–11)	**0.006**
	(Sub)cutaneous	2.7 (0–6)	3.8 (1–6)	**0.013**
	Deep	1.7 (0–4)	2.8 (1–5)	**0.002**

The table shows the mean number of Pacinian corpuscles, with ranges in parentheses, for distinct localizations in the forefoot of patients with diabetic sensorimotor polyneuropathy (DSP) compared to healthy volunteers

*DSP* diabetic sensorimotor polyneuropathy, *MTP* metatarsophalangeal

^a^ *p*-value for comparison of Pacinian corpuscle number in DSP patients and healthy volunteers by Wilcoxon signed-rank test. Significant results (*p* < 0.05) are written in bold

**Fig. 6:**
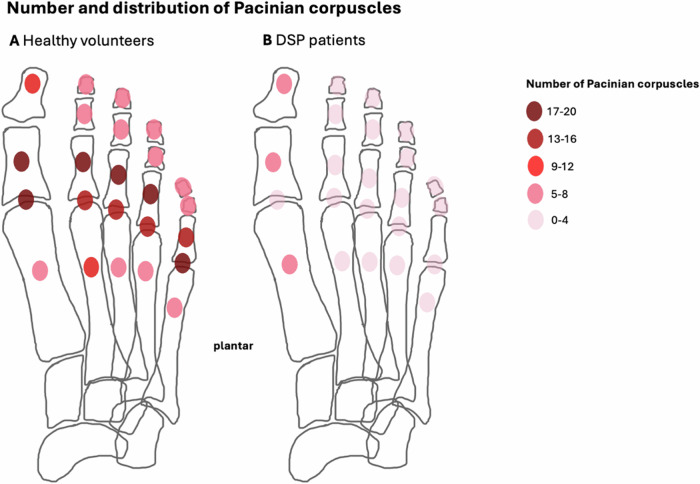
Schematic illustration of numbers of Pacinian corpuscles (PC) in the plantar side of the forefoot of healthy volunteers (**A**) and patients with diabetic sensorimotor polyneuropathy (DSP) (**B**). The number of PC is indicated by the color intensity illustrated in the figure legend. In healthy volunteers, the locations with the most tightly clustered PC were along the proximal phalanges, followed by the metatarsophalangeal joints and the distal phalanges. In contrast, the overall number of PC in DSP patients was significantly reduced, and no predominance for an individual location was found

Further, the typical “chain-like” PC pattern in healthy volunteers with focal clustering was almost completely disrupted in DSP patients. Instead, MR imaging revealed an irregular, heterogeneous arrangement with fewer and more isolated localized PC, which we termed a “spot-like” pattern (Figs. 4 and 5). Also, there was a different frequency distribution of PC regarding distinct subregions of the forefoot in DSP patients compared to healthy volunteers: In healthy volunteers, quantitative analysis revealed the highest number of PC along the proximal phalanges, followed by the MTP joints, the distal phalanges, the metatarsal bones, and the middle phalanges (Table 3, Fig. 6). In DSP patients, substantially fewer PC were present, without a clear predominance for any anatomical subregion.

The maximum PC diameter in DSP patients was 3 mm (range 1–3 mm), while that of healthy volunteers was 5 mm (range 1–5 mm).

### Inter- and intra-reader agreement

The inter-reader agreement ranged between substantial to almost perfect for assessing PC numbers in healthy volunteers (ICC range: 0.618–0.971) and DSP patients (ICC range: 0.761–1.000), depending on the anatomical subregion. The intra-reader agreement for reader 1 ranged between substantial to almost perfect for assessing PC numbers in volunteers (ICC range: 0.748–1.000) and moderate to almost perfect for assessing PC numbers in DSP patients (ICC range: 0.579–1.000). The intra-reader agreement for reader 2 was almost perfect for assessing PC numbers in volunteers (ICC range: 0.850–1.000) and substantial to almost perfect for assessing PC numbers in DSP patients (ICC range: 0.626–1.000). Detailed ICC values, including 95% CI, are provided in Supplementary Tables 1–3.

### Association between Pacinian corpuscle number and clinical parameters

In the DSP cohort, the mean duration of diabetes was 234.8 ± 130.4 months, and the mean HbA1c was 7.6 ± 1.1%. Neither the duration of type 2 diabetes nor HbA1c values correlated with the PC number, as calculated for the total number of PC along each digit I-V and the forefoot as a whole, respectively (duration of type 2 diabetes: Spearman’s ρ range = −0.335 to −0.133; *p*-value range = 0.15 to 0.58 and HbA1c: Spearman’s ρ range = −0.151 to 0.055; *p*-value range = 0.52 to 0.89). Detailed results are provided in Supplementary Table 4.

## Discussion

This study revealed MRI findings of patients with type 2 diabetes-derived sensorimotor polyneuropathy (DSP): Patients with DSP had significantly lower numbers of Pacinian corpuscles (PC) in the forefoot compared to healthy volunteers matched for age and gender. Furthermore, in patients with DSP, PC featured a sparse “spot-like” pattern rather than the typical “chain-like” configuration in healthy volunteers and were smaller in size.

DSP is the most common type of neuropathy and is a leading cause of morbidity and mortality in diabetic patients. Therefore, it represents a considerable burden for the individual patient and the healthcare system [4–6, 13]. Delays in the diagnosis of DSP may contribute to the manifestation of diabetic foot syndrome with symptoms such as numbness, tingling, and loss of feeling, potentially leading to the development of severe ulcerations and amputation of the foot [23]. Besides clinical examination, various quantitative tests based on functional and structural assessment of intraepidermal nerve fibers, such as quantitative sensory testing and skin biopsy, are available to diagnose DSP and determine its severity [7–11]. Previous immunochemistry studies demonstrated that cutaneous mechanoreceptors, including PC, are altered regarding their topography, morphology, and structure in diabetic conditions [4, 13]. In addition, PC can be well visualized with high-resolution ultrasound and MRI [14–17]. Yet, this was the first study that assessed PC in diabetic patients using MRI.

The most relevant finding of our study was that in DSP patients, the number of PC on forefoot MRI was significantly reduced compared to healthy volunteers (82.7 ± 46.1 vs. 265.3 ± 49.3). This was true for the overall number of PC, each digit, and distinct subregions. Analyzing distinct subregions, we found the lowest overall number of PC in healthy volunteers to be *n* = 9 along the second to fifth MTP joint, whereas the highest number in DSP patients was *n* = 6. This means the second to fifth MTP joint subregion did not overlap in PC numbers between DSP patients and healthy volunteers. Therefore, it could be an area of interest when analyzing forefoot MR examinations of individuals with suspected DSP. Consequently, for daily practice, we recommend considering reduced PC numbers on forefoot MRI as suspicious for DSP if there are less than *n* = 9 PC along the second to fifth MTP joint, each considered individually. Alternatively, a threshold of less than *n* = 180 PC for the whole forefoot could be used, but counting the PC along one of the MTP joints II-V is more applicable for daily routine.

Beyond that, a “spot-like” PC distribution pattern was detected in DSP patients, rather than the typical “chain-like” arrangement of PC in healthy individuals. In line with Germann et al, who observed the highest numbers of PC at the plantar side of the MTP joints and proximal phalanges in two healthy individuals at 7-T MRI [14], in our study with 3-T MRI, quantitative analysis revealed the highest numbers of PC in healthy volunteers along the MTP joints, followed by the proximal and distal phalanges. In contrast, no clear predominance for any anatomical area was seen for the PC distribution in DSP patients. Besides, in DSP patients, PC were smaller, ranging from a diameter of 1–3 mm, while in healthy volunteers, the maximum PC size was up to 5 mm. This underlines that in DSP, PC are not only affected topographically regarding their arrangement but also morphologically regarding their size.

For everyday clinical practice, these results might imply that patients who show a reduced number and conspicuous distribution pattern of PC on forefoot MRI might benefit from initiating further workup, including neurologic examination and HbA1c testing, as a next step to detect those who suffer from previously unknown diabetes.

No correlation was found when analyzing associations between the number of PC and clinical parameters, including the duration of type 2 diabetes and HbA1c values. However, this could be because our cohort may not have been large enough to detect such a correlation.

Several limitations of this study need to be addressed. First, this was a single-center study with a partly retrospective design and a limited number of patients, wherefore a potential selection bias might have occurred. In addition, due to heterogeneous documentation, it was impossible to reliably compare differences in the severity of neuropathy between patients. However, retrospectively selected DSP patients were matched for age and gender with healthy volunteers to increase study efficiency and reduce potential confounding effects. Second, as patients were retrospectively selected, no histopathological correlation was available. However, we can confidently assume the correct identification of PC based on previous studies with anatomic/histologic correlation [16, 24, 25] and a clear depiction of their typical nodular appearance instead of linear branching vessels. Yet, due to different scanners for patients and healthy volunteers, potential variability in PC detection needs to be acknowledged.

In conclusion, this study evaluated Pacinian corpuscles (PC) on forefoot MRI of patients with type 2 diabetes-derived sensorimotor polyneuropathy (DSP) and demonstrated that DSP patients have significantly lower PC numbers than healthy volunteers and feature a “spot-like” pattern of PC distribution instead of the typical “chain-like” configuration. These findings may open the possibility of using MRI as a noninvasive diagnostic tool for assessing patients with DSP. Yet, the clinical value of this diagnostic approach needs to be further solidified in future multi-center studies analyzing the association between DSP progression and changes in PC numbers and distribution in the longitudinal course.

## Supplementary information

ELECTRONIC SUPPLEMENTARY MATERIAL

## Data Availability

The data analyzed during this study are available from the corresponding author upon reasonable request.

## Abbreviations

DSP: Diabetic sensorimotor polyneuropathy
FS: Fat-saturated
HbA1c: Hemoglobin A1c
ICC: Intraclass correlation coefficient
MTP: Metatarsophalangeal
PACS: Picture Archiving and Communication System
PC: Pacinian corpuscle(s)
PD: Proton-density
TSE: Turbo spin-echo
