# Supplementary material for: MRI evaluation of Pacinian corpuscle number and distribution in the forefoot in diabetic sensorimotor polyneuropathy
Source: Insights Imaging. 2025 Mar 7;16:52. doi: 10.1186/s13244-025-01932-8 (PMC11889331; doi:10.1186/s13244-025-01932-8)
Supplement: Supplementary file 1 — ELECTRONIC SUPPLEMENTARY MATERIAL [file 13244_2025_1932_MOESM1_ESM.pdf]

MRI evaluation of Pacinian corpuscle number and distribution in the forefoot in diabetic sensorimotor polyneuropathy

ELECTRONIC SUPPLEMENTARY MATERIAL

Supplementary Table 1: Inter-reader agreement.

			ICC *	Lower 95% CL	Upper 95% CL
**Healthy volunteers**					
**Digit I**	Distal phalanx	*(Sub)cutaneous*	0.966	0.916	0.987
		Deep	0.971	0.918	0.989
	Proximal phalanx	*(Sub)cutaneous*	0.910	0.756	0.965
		Deep	0.963	0.909	0.985
	MTP joint	*(Sub)cutaneous*	0.965	0.896	0.987
		Deep	0.938	0.835	0.976
	Metatarsal bone	*(Sub)cutaneous*	0.792	0.367	0.924
		Deep	0.879	0.698	0.952
**Digit II**	Distal phalanx	*(Sub)cutaneous*	0.618	0.034	0.850
		Deep	0.889	0.688	0.958
	Middle phalanx	*(Sub)cutaneous*	0.807	0.484	0.925
		Deep	0.634	0.061	0.857
	Proximal phalanx	*(Sub)cutaneous*	0.957	0.893	0.983
		Deep	0.716	0.285	0.887
	MTP joint	*(Sub)cutaneous*	0.954	0.881	0.982
		Deep	0.873	0.682	0.950
	Metatarsal bone	*(Sub)cutaneous*	0.922	0.750	0.972
		Deep	0.772	0.438	0.909
**Digit III**	Distal phalanx	*(Sub)cutaneous*	0.841	0.135	0.953
		Deep	0.842	0.595	0.938
	Middle phalanx	*(Sub)cutaneous*	0.900	0.731	0.961
		Deep	0.814	0.495	0.929
	Proximal phalanx	*(Sub)cutaneous*	0.921	0.803	0.968
		Deep	0.902	0.682	0.965
	MTP joint	*(Sub)cutaneous*	0.806	0.512	0.923
		Deep	0.920	0.797	0.968
	Metatarsal bone	*(Sub)cutaneous*	0.829	0.346	0.942
		Deep	0.913	0.657	0.971
**Digit IV**	Distal phalanx	*(Sub)cutaneous*	0.865	0.642	0.948
		Deep	0.830	0.577	0.933
	Middle phalanx	*(Sub)cutaneous*	0.819	0.534	0.929
		Deep	0.847	0.618	0.939
	Proximal phalanx	*(Sub)cutaneous*	0.958	0.894	0.983
		Deep	0.876	0.645	0.953
	MTP joint	*(Sub)cutaneous*	0.885	0.716	0.954
		Deep	0.936	0.839	0.975
	Metatarsal bone	*(Sub)cutaneous*	0.893	0.731	0.958
		Deep	0.903	0.743	0.962

**Digit V**	Distal phalanx	*(Sub)cutaneous*	0.854	0.607	0.943
		Deep	0.810	0.455	0.928
	Middle phalanx	*(Sub)cutaneous*	0.818	0.474	0.932
		Deep	0.747	0.343	0.901
	Proximal phalanx	*(Sub)cutaneous*	0.940	0.795	0.979
		Deep	0.811	0.517	0.926
	MTP joint	*(Sub)cutaneous*	0.868	0.671	0.947
		Deep	0.869	0.672	0.948
	Metatarsal bone	*(Sub)cutaneous*	0.878	0.571	0.957
		Deep	0.896	0.475	0.967
**DSP patients**			**ICC ***	**Lower 95% CL**	**Upper 95% CL**
**Digit I**	Distal phalanx	*(Sub)cutaneous*	0.930	0.827	0.972
		Deep	0.980	0.949	0.992
	Proximal phalanx	*(Sub)cutaneous*	0.939	0.846	0.976
		Deep	0.945	0.773	0.982
	MTP joint	*(Sub)cutaneous*	0.966	0.848	0.989
		Deep	0.976	0.939	0.990
	Metatarsal bone	*(Sub)cutaneous*	0.983	0.956	0.993
		Deep	0.955	0.885	0.982
**Digit II**	Distal phalanx	*(Sub)cutaneous*	0.951	0.867	0.981
		Deep	0.948	0.748	0.984
	Middle phalanx	*(Sub)cutaneous*	0.951	0.877	0.981
		Deep	0.872	0.683	0.949
	Proximal phalanx	*(Sub)cutaneous*	0.962	0.899	0.985
		Deep	0.942	0.842	0.977
	MTP joint	*(Sub)cutaneous*	0.892	0.724	0.957
		Deep	0.860	0.648	0.944
	Metatarsal bone	*(Sub)cutaneous*	0.967	0.917	0.987
		Deep	0.924	0.809	0.970
**Digit III**	Distal phalanx	*(Sub)cutaneous*	0.961	0.903	0.984
		Deep	1.000	1.000	1.000
	Middle phalanx	*(Sub)cutaneous*	0.959	0.897	0.984
		Deep	0.938	0.750	0.979
	Proximal phalanx	*(Sub)cutaneous*	0.961	0.902	0.984
		Deep	0.934	0.831	0.974
	MTP joint	*(Sub)cutaneous*	0.958	0.895	0.983
		Deep	0.867	0.666	0.947
	Metatarsal bone	*(Sub)cutaneous*	0.973	0.931	0.989
		Deep	0.964	0.907	0.986
**Digit IV**	Distal phalanx	*(Sub)cutaneous*	0.947	0.870	0.979
		Deep	0.903	0.676	0.965
	Middle phalanx	*(Sub)cutaneous*	1.000	1.000	1.000
		Deep	0.761	0.306	0.911
	Proximal phalanx	*(Sub)cutaneous*	0.951	0.822	0.983
		Deep	0.930	0.747	0.976
	MTP joint	*(Sub)cutaneous*	0.951	0.875	0.980
		Deep	0.876	0.674	0.951
	Metatarsal bone	*(Sub)cutaneous*	0.966	0.873	0.988
		Deep	0.964	0.881	0.987
**Digit V**	Distal phalanx	*(Sub)cutaneous*	0.965	0.867	0.988
		Deep	0.914	0.667	0.971

	Middle phalanx	*(Sub)cutaneous*	0.846	0.512	0.944
		Deep	0.875	0.600	0.955
	Proximal phalanx	*(Sub)cutaneous*	0.897	0.644	0.964
		Deep	0.963	0.899	0.986
	MTP joint	*(Sub)cutaneous*	0.941	0.641	0.982
		Deep	0.875	0.600	0.955
	Metatarsal bone	*(Sub)cutaneous*	0.972	0.926	0.989
		Deep	0.965	0.875	0.988

***** The level of agreement was categorized as follows [19]: .0=poor, .01-.20=slight, .21-.40=fair, .41-.60=moderate, .61-.80=substantial, .81-1.00=almost perfect agreement.
CL, confidence limit; DSP, diabetic sensorimotor polyneuropathy; ICC, intraclass correlation coefficient; MTP, metatarsophalangeal.

Supplementary Table 2: Intra-reader agreement Reader 1.

			ICC *	Lower 95% CL	Upper 95% CL
**Healthy volunteers**					
**Digit I**	Distal phalanx	*(Sub)cutaneous*	0.896	0.358	0.970
		Deep	0.883	0.704	0.954
	Proximal phalanx	*(Sub)cutaneous*	0.940	0.852	0.976
		Deep	0.966	0.914	0.986
	MTP joint	*(Sub)cutaneous*	0.953	0.827	0.984
		Deep	0.941	0.854	0.977
	Metatarsal bone	*(Sub)cutaneous*	1.000	1.000	1.000
		Deep	0.926	0.569	0.978
**Digit II**	Distal phalanx	*(Sub)cutaneous*	0.841	0.596	0.937
		Deep	0.843	0.602	0.938
	Middle phalanx	*(Sub)cutaneous*	0.827	0.573	0.931
		Deep	0.919	0.799	0.968
	Proximal phalanx	*(Sub)cutaneous*	0.979	0.947	0.992
		Deep	0.899	0.729	0.961
	MTP joint	*(Sub)cutaneous*	0.968	0.921	0.987
		Deep	0.956	0.782	0.986
	Metatarsal bone	*(Sub)cutaneous*	0.959	0.690	0.988
		Deep	0.839	0.599	0.936
**Digit III**	Distal phalanx	*(Sub)cutaneous*	0.934	0.835	0.974
		Deep	0.898	0.739	0.960
	Middle phalanx	*(Sub)cutaneous*	0.826	0.571	0.931
		Deep	0.841	0.593	0.937
	Proximal phalanx	*(Sub)cutaneous*	0.956	0.813	0.985
		Deep	0.913	0.780	0.966
	MTP joint	*(Sub)cutaneous*	0.857	0.637	0.943
		Deep	0.927	0.813	0.971
	Metatarsal bone	*(Sub)cutaneous*	0.900	0.753	0.960
		Deep	0.921	0.785	0.970
**Digit IV**	Distal phalanx	*(Sub)cutaneous*	0.921	0.785	0.970
		Deep	0.835	0.373	0.944
	Middle phalanx	*(Sub)cutaneous*	0.748	0.381	0.899
		Deep	0.942	0.837	0.978
	Proximal phalanx	*(Sub)cutaneous*	0.961	0.902	0.985
		Deep	0.961	0.902	0.985
	MTP joint	*(Sub)cutaneous*	0.891	0.722	0.957
		Deep	0.930	0.810	0.973
	Metatarsal bone	*(Sub)cutaneous*	0.935	0.838	0.974
		Deep	0.944	0.860	0.978
**Digit V**	Distal phalanx	*(Sub)cutaneous*	0.914	0.782	0.966
		Deep	0.942	0.853	0.977
	Middle phalanx	*(Sub)cutaneous*	0.853	0.625	0.942
		Deep	0.788	0.470	0.916
	Proximal phalanx	*(Sub)cutaneous*	0.970	0.812	0.991
		Deep	0.946	0.865	0.979
	MTP joint	*(Sub)cutaneous*	0.927	0.818	0.971
		Deep	0.934	0.834	0.974
	Metatarsal bone	*(Sub)cutaneous*	0.943	0.856	0.978
		Deep	0.951	0.873	0.981
**DSP patients**			**ICC ***	**Lower 95% CL**	**Upper 95% CL**
**Digit I**	Distal phalanx	*(Sub)cutaneous*	0.946	0.864	0.978
		Deep	0.971	0.926	0.989
	Proximal phalanx	*(Sub)cutaneous*	0.960	0.901	0.984
		Deep	0.963	0.801	0.989

	MTP joint	*(Sub)cutaneous*	0.980	0.949	0.992
		Deep	0.980	0.913	0.993
	Metatarsal bone	*(Sub)cutaneous*	0.965	0.909	0.986
		Deep	0.974	0.933	0.990
**Digit II**	Distal phalanx	*(Sub)cutaneous*	0.953	0.872	0.982
		Deep	0.896	0.606	0.965
	Middle phalanx	*(Sub)cutaneous*	0.922	0.754	0.971
		Deep	0.856	0.406	0.953
	Proximal phalanx	*(Sub)cutaneous*	0.929	0.604	0.978
		Deep	0.930	0.611	0.979
	MTP joint	*(Sub)cutaneous*	0.854	0.315	0.954
		Deep	0.959	0.898	0.984
	Metatarsal bone	*(Sub)cutaneous*	0.969	0.863	0.990
		Deep	0.927	0.803	0.972
**Digit III**	Distal phalanx	*(Sub)cutaneous*	0.950	0.752	0.984
		Deep	0.960	0.857	0.986
	Middle phalanx	*(Sub)cutaneous*	0.992	0.980	0.997
		Deep	0.862	0.445	0.954
	Proximal phalanx	*(Sub)cutaneous*	0.949	0.808	0.982
		Deep	0.850	0.626	0.940
	MTP joint	*(Sub)cutaneous*	0.977	0.943	0.991
		Deep	0.799	0.501	0.920
	Metatarsal bone	*(Sub)cutaneous*	0.966	0.886	0.988
		Deep	0.946	0.824	0.981
**Digit IV**	Distal phalanx	*(Sub)cutaneous*	0.953	0.791	0.984
		Deep	0.808	0.486	0.926
	Middle phalanx	*(Sub)cutaneous*	1.000	1.000	1.000
		Deep	0.775	0.280	0.919
	Proximal phalanx	*(Sub)cutaneous*	0.916	0.622	0.973
		Deep	0.904	0.628	0.967
	MTP joint	*(Sub)cutaneous*	0.956	0.879	0.983
		Deep	0.866	0.649	0.947
	Metatarsal bone	*(Sub)cutaneous*	0.970	0.910	0.989
		Deep	0.988	0.969	0.995
**Digit V**	Distal phalanx	*(Sub)cutaneous*	0.947	0.862	0.979
		Deep	0.871	0.529	0.955
	Middle phalanx	*(Sub)cutaneous*	0.888	0.524	0.963
		Deep	0.873	0.679	0.950
	Proximal phalanx	*(Sub)cutaneous*	0.856	0.601	0.945
		Deep	0.900	0.685	0.964
	MTP joint	*(Sub)cutaneous*	0.962	0.906	0.985
		Deep	0.579	0.009	0.828
	Metatarsal bone	*(Sub)cutaneous*	0.956	0.839	0.985
		Deep	0.981	0.953	0.993

***** The level of agreement was categorized as follows [19]: .0=poor, .01-.20=slight, .21-.40=fair, .41-.60=moderate, .61-.80=substantial, .81-1.00=almost perfect agreement.
CL, confidence limit; DSP, diabetic sensorimotor polyneuropathy; ICC, intraclass correlation coefficient; MTP, metatarsophalangeal.

Supplementary Table 3: Intra-reader agreement Reader 2.

			ICC *	Lower 95% CL	Upper 95% CL
**Healthy volunteers**					
**Digit I**	Distal phalanx	*(Sub)cutaneous*	0.941	0.781	0.980
		Deep	0.926	0.815	0.970
	Proximal phalanx	*(Sub)cutaneous*	0.950	0.851	0.981
		Deep	0.979	0.947	0.991
	MTP joint	*(Sub)cutaneous*	0.918	0.796	0.967
		Deep	0.943	0.857	0.977
	Metatarsal bone	*(Sub)cutaneous*	0.958	0.890	0.983
		Deep	0.952	0.881	0.981
**Digit II**	Distal phalanx	*(Sub)cutaneous*	0.938	0.844	0.975
		Deep	0.974	0.934	0.990
	Middle phalanx	*(Sub)cutaneous*	0.940	0.489	0.984
		Deep	0.886	0.718	0.955
	Proximal phalanx	*(Sub)cutaneous*	0.963	0.908	0.985
		Deep	0.949	0.873	0.980
	MTP joint	*(Sub)cutaneous*	0.968	0.921	0.987
		Deep	0.882	0.707	0.953
	Metatarsal bone	*(Sub)cutaneous*	0.949	0.872	0.980
		Deep	0.972	0.929	0.989
**Digit III**	Distal phalanx	*(Sub)cutaneous*	0.894	0.736	0.958
		Deep	0.950	0.873	0.980
	Middle phalanx	*(Sub)cutaneous*	0.950	0.873	0.980
		Deep	0.925	0.813	0.970
	Proximal phalanx	*(Sub)cutaneous*	0.974	0.933	0.990
		Deep	0.967	0.917	0.987
	MTP joint	*(Sub)cutaneous*	0.880	0.695	0.953
		Deep	0.959	0.896	0.984
	Metatarsal bone	*(Sub)cutaneous*	0.948	0.870	0.979
		Deep	0.931	0.755	0.976
**Digit IV**	Distal phalanx	*(Sub)cutaneous*	0.941	0.852	0.977
		Deep	0.932	0.830	0.973
	Middle phalanx	*(Sub)cutaneous*	0.850	0.618	0.941
		Deep	0.947	0.867	0.979
	Proximal phalanx	*(Sub)cutaneous*	0.991	0.977	0.996
		Deep	0.991	0.978	0.996
	MTP joint	*(Sub)cutaneous*	0.952	0.711	0.985
		Deep	0.885	0.713	0.954
	Metatarsal bone	*(Sub)cutaneous*	0.955	0.884	0.982
		Deep	0.925	0.811	0.971
**Digit V**	Distal phalanx	*(Sub)cutaneous*	0.984	0.961	0.994
		Deep	0.926	0.812	0.971
	Middle phalanx	*(Sub)cutaneous*	0.929	0.819	0.972
		Deep	0.884	0.713	0.954
	Proximal phalanx	*(Sub)cutaneous*	0.981	0.952	0.992
		Deep	0.957	0.892	0.983
	MTP joint	*(Sub)cutaneous*	0.915	0.786	0.966
		Deep	0.892	0.700	0.959
	Metatarsal bone	*(Sub)cutaneous*	0.946	0.866	0.978
		Deep	0.919	0.788	0.969
**DSP patients**			**ICC ***	**Lower 95% CL**	**Upper 95% CL**
**Digit I**	Distal phalanx	*(Sub)cutaneous*	0.889	0.717	0.956
		Deep	0.978	0.943	0.991
	Proximal phalanx	*(Sub)cutaneous*	0.942	0.854	0.977
		Deep	0.958	0.892	0.984

	MTP joint	*(Sub)cutaneous*	0.959	0.783	0.987
		Deep	0.961	0.884	0.986
	Metatarsal bone	*(Sub)cutaneous*	0.981	0.953	0.993
		Deep	0.985	0.961	0.994
**Digit II**	Distal phalanx	*(Sub)cutaneous*	0.951	0.875	0.980
		Deep	0.941	0.699	0.981
	Middle phalanx	*(Sub)cutaneous*	0.923	0.692	0.974
		Deep	0.897	0.744	0.959
	Proximal phalanx	*(Sub)cutaneous*	0.971	0.903	0.990
		Deep	0.906	0.766	0.963
	MTP joint	*(Sub)cutaneous*	0.754	0.394	0.902
		Deep	0.955	0.882	0.982
	Metatarsal bone	*(Sub)cutaneous*	0.951	0.879	0.981
		Deep	0.922	0.791	0.970
**Digit III**	Distal phalanx	*(Sub)cutaneous*	0.957	0.882	0.983
		Deep	0.898	0.747	0.960
	Middle phalanx	*(Sub)cutaneous*	0.884	0.640	0.957
		Deep	0.927	0.789	0.973
	Proximal phalanx	*(Sub)cutaneous*	0.955	0.883	0.982
		Deep	1.000	1.000	1.000
	MTP joint	*(Sub)cutaneous*	0.858	0.649	0.943
		Deep	0.828	0.576	0.931
	Metatarsal bone	*(Sub)cutaneous*	0.990	0.974	0.996
		Deep	0.934	0.833	0.974
**Digit IV**	Distal phalanx	*(Sub)cutaneous*	0.948	0.811	0.982
		Deep	0.795	0.495	0.918
	Middle phalanx	*(Sub)cutaneous*	0.960	0.895	0.984
		Deep	0.626	0.109	0.848
	Proximal phalanx	*(Sub)cutaneous*	0.968	0.915	0.987
		Deep	0.941	0.810	0.979
	MTP joint	*(Sub)cutaneous*	0.963	0.907	0.985
		Deep	0.858	0.631	0.945
	Metatarsal bone	*(Sub)cutaneous*	0.965	0.885	0.988
		Deep	0.933	0.827	0.974
**Digit V**	Distal phalanx	*(Sub)cutaneous*	0.977	0.943	0.991
		Deep	1.000	1.000	1.000
	Middle phalanx	*(Sub)cutaneous*	0.920	0.797	0.968
		Deep	0.872	0.683	0.949
	Proximal phalanx	*(Sub)cutaneous*	0.872	0.680	0.949
		Deep	0.924	0.809	0.970
	MTP joint	*(Sub)cutaneous*	0.962	0.905	0.985
		Deep	0.755	0.342	0.906
	Metatarsal bone	*(Sub)cutaneous*	0.958	0.895	0.983
		Deep	0.887	0.719	0.955

***** The level of agreement was categorized as follows [19]: .0=poor, .01-.20=slight, .21-.40=fair, .41-.60=moderate, .61-.80=substantial, .81-1.00=almost perfect agreement.
CL, confidence limit; DSP, diabetic sensorimotor polyneuropathy; ICC, intraclass correlation coefficient; MTP, metatarsophalangeal.

Supplementary Table 4: Associations between the number of Pacinian corpuscles and clinical parameters.

**PC number and duration of DM**	Spearman's ρ	*P*-value
Digit I	-0.160	.50
Digit II	-0.133	.58
Digit III	-0.289	.22
Digit IV	-0.335	.15
Digit V	-0.258	.27
Digit I-V	-0.271	.25
**PC number and HbA1c value**		
Digit I	0.055	.82
Digit II	-0.033	.89
Digit III	-0.085	.72
Digit IV	-0.151	.52
Digit V	-0.134	.57
Digit I-V	-0.037	.88

DM, diabetes mellitus (type 2); PC, Pacinian corpuscle.
